# Rapid On-Site NIR Spectroscopic Characterization of CWA Liquids Using a Novel 3D-Printed Glass Cell

**DOI:** 10.1021/acs.analchem.5c03719

**Published:** 2025-09-16

**Authors:** Jelle C. de Koning, Marcel J. van der Schans, Lai Fun Chau, Tom Venema, Gert IJ. Salentijn, Saer Samanipour, Gertjan Bon, Henk-Jan Ramaker, Teun van Wieringen, Jos Oomens, Arian C. van Asten

**Affiliations:** a Dep. CBRN Protection, TNO Defence Safety and Security, Lange Kleiweg 137, Rijswijk 2288 GJ, The Netherlands; b Van't Hoff Institute for Molecular Sciences, Faculty of Science, 84709 University of Amsterdam, P.O. Box 94157, Amsterdam 1090 GD, The Netherlands; c CLHC, Netherlands Center for Forensic Science and Medicine, 84709 University of Amsterdam, P.O. Box 94157, Amsterdam 1090 GD, The Netherlands; d Laboratory of Organic Chemistry, 4508 Wageningen University & Research, Stippeneng 4, Wageningen 6708 WE, The Netherlands; e Wageningen Food Safety Research, P.O. Box 230, Wageningen 6700 AE, The Netherlands; f Technology Centre, Faculty of Science, 84709 University of Amsterdam, P.O. Box 94157, Amsterdam 1090 GD, The Netherlands; g TIPb, Wilhelminaplein 30, Amsterdam 1062 KR, The Netherlands; h FELIX Laboratory, Institute for Molecules and Materials, 6029 Radboud University, Toernooiveld 7, Nijmegen 6525 ED, The Netherlands

## Abstract

After an incident with suspected use of chemical warfare agents (CWAs) has occurred, fast and reliable detection and identification are pivotal to take the right actions and limit the afflicted damage. Current available methodologies face practical, selectivity, or sensitivity limitations. Near-infrared spectroscopy (NIRS) has been proven to be suitable for on-site analysis in various fields, but it is known to be challenging for the spectral analysis of liquids. The current study introduces a 3D-printed glass liquid cell that overcomes practical limitations of on-site NIR analysis of liquid samples while providing a single-use container for safe sampling and analysis of potentially very toxic compounds. The liquid cell can be implemented for most NIR detectors and is suitable for the analysis, storage, and transport of samples of interest. Validation experiments show that the cell can be manufactured and used in a reproducible manner and has no catalytic effect on sample degradation. The final design is applied to safely record information-rich spectra of several CWAs using two different NIR detectors, a high resolution benchtop laboratory instrument, and a small, rapid, portable device. Recorded spectra contain sufficient compound-specific information to distinguish between different classes of CWAs and different agents within the same class. Results from both instruments are in good agreement and comparable to theoretically predicted spectra and thus show the applicability of NIR spectroscopy to the analysis of CWAs. It is expected that the developed liquid cell is also useful for NIR analysis of liquids in other fields.

## Graphical abstract

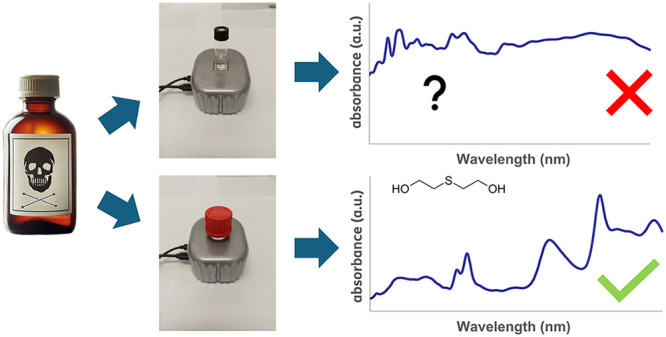

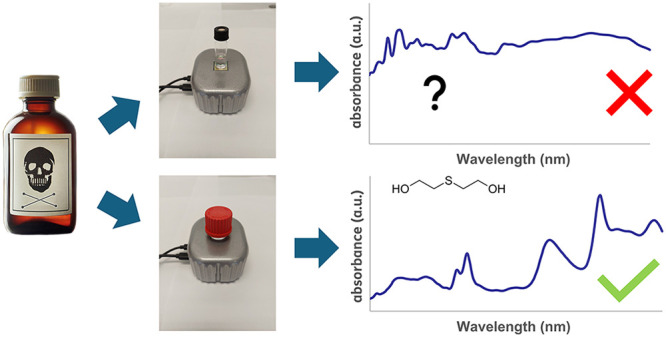

Chemical warfare agents (CWAs) are extremely toxic chemicals that have been used and undergone further development for over a century.^1^ Despite international efforts to completely ban the use of these chemicals, nerve agents have been deployed in both assassinations and in large scale attacks in the past decades.^2–4^ Many different verification methods have been established to identify CWAs. During inspections and incident investigations, chemicals can ideally be tentatively identified in the field before the samples are sent to a dedicated laboratory for confirmation. Due to the extreme toxicity of these chemicals, with deadly doses in the microgram per kilogram body mass range, it is of utmost importance to rapidly identify them in a situation of suspected use and exposure. After identification, precautions are taken to avoid further contact. In addition, correct countermeasures can be taken.

There are recommended operation procedures on how to detect CWAs in off-site laboratories. Different methods are also available for the on-site detection of these chemicals including ion mobility spectrometry (IMS), portable mass spectrometry, possibly preceded by a mobile separation technique such as gas chromatography, and different spectroscopic techniques.^5–9^ While these techniques are powerful in identifying chemicals, practical limitations in on-site analysis remain when dealing with toxic chemicals of low volatility. Spectroscopic techniques typically allow for the analysis of samples in a noninvasive and contact-free manner. Developments have also allowed for the analysis of samples from a short distance away from the sample using spectroscopic techniques. Both Raman and infrared spectroscopy have been successfully used for the detection of CWAs or CWA simulants in stand-off and point detection modes.^5,9–13^ To the best of our knowledge, the use of near-infrared spectroscopy (NIRS) for CWA analysis has not been reported so far.

NIR spectroscopy has several practical advantages. Analysis typically takes seconds, while other spectroscopic techniques such as Raman spectroscopy may require minutes for challenging samples. In addition, NIR allows for extensive miniaturization, and as a result, small, portable NIR detectors have been commercially available for some time. Compared to other portable spectroscopic techniques such as FT-IR and Raman, NIR detectors are lower in costs and generally smaller and more robust. In recent years, portable NIR has been successfully applied to detect and identify explosives and drugs of abuse.^15–19^ NIR is ideally suited for the characterization of sensitive and reactive materials because of the low energy of the radiation used to interrogate the samples. Hence, samples do not react because of light exposure and are fully preserved for follow-up analysis in the laboratory. Although light exposure can cause some sample heating, this makes NIR spectroscopy ideally suited for CWA characterization.

Analysis of solid evidence materials is typically done in diffuse reflectance mode, where part of the light is reflected back to the detector after interaction with the sample.^20,21^ This mode of analysis is most often used as detectors are typically designed to analyze solid sample materials.^22^ In their pure form, most CWAs are transparent, colorless liquids, making diffuse reflectance measurements insensitive, as no light is reflected back to the detector. Tailored approaches are needed when dealing with liquids in diffuse reflectance mode. Such approaches have been previously investigated; furthermore, a more classical transmission measurement can successfully be used for the analysis of liquid samples.^22–24^ While these approaches have been shown to yield informative NIR spectra of liquids, most systems are either open, with the risk of coming into contact with the sample or vapors, or require altering of the sample by addition of a reflecting agent. As for CWA-related investigations, the chemicals of interest are extremely toxic, and sample integrity is of great importance; previously described solutions do not meet the stringent requirements needed for this application.

One way of working with dangerous chemicals is by employing sealable and inert glass containers. Glass containers have extensively been used in studies using NIR spectroscopy, as glass is NIR-transparent and does not show any significant absorption over the entire NIR wavelength range. Consequently, a sample can be analyzed without taking it out of the container.^25^ For solid samples, regular glass sample vials will suffice for most NIR instruments. However, using glass containers for the NIR characterization of liquids under safe conditions will require tailor-made solutions. Traditionally, custom-built glassware for special applications can be created by glass blowing at high temperatures.

Recent developments in the fields of 3D printing and glass processing allow for the 3D printing of glass using a technique called digital light processing (DLP).^26,27^ In analytical sciences, 3D printing has been extensively used for prototyping. The use of 3D printed glass is, however, far less studied. One of the main advantages of 3D printing compared to manual processing is the high reproducibility of the end product. Utilizing this technique, containers can be made in a specific shape suitable for NIR analysis of liquids of interest. In order to reflect light in NIR spectroscopy, polytetrafluoroethylene (PTFE) is a suitable candidate as it can fully reflect light based on the surface roughness.^28^ PTFE reportedly has a high chemical inertness, making it unlikely to react to any chemicals it comes in contact with.^29^ Besides the reflective properties of PTFE, its inertness makes it a suitable material to be used in sample storage. When a sample is added to a container with PTFE parts, direct contact will not affect the sample of interest, thereby enabling further analysis.

In transmission mode, the overall absorbance at a given NIR wavelength is directly proportional to the path length. The desired path length is determined by molecular properties and are therefore compound specific.^30^ Optimizing this value is important especially in situations where compounds of interest are present in relative low concentration and maximum sensitivity is needed, such as in aqueous solutions.^31^ However, for relatively pure compounds (such as those analyzed in this study), it is important that spectral details are conserved to maintain and maximize selectivity in order to enable a robust and trustworthy identification. To that end, excessive absorption due to an extensive effective path length must be prevented.

In this study, we introduce a liquid-cell design that can be safely used for the acquisition of NIR spectra of extremely dangerous liquids. The liquid cell was designed as a small glass 3D-printed device with PTFE as reflecting material due to its practical advantages. A small path length of 0.5 mm was chosen to allow for sufficient sensitivity and spectral detail while using small sample volumes. The path length is determined by the thickness of a PTFE spacer that also acts as a seal to contain the sample. The proposed design is depicted in Figure 1. The device is sealable, making it suitable for the analysis and transport of dangerous chemicals. The proposed solution does not require the addition of any material to the sample, making it suitable for the analysis of samples that require preservation. The production costs are sufficiently low for single use operation. The final design was evaluated for safety, practical use and reproducibility. Using this liquid cell, the applicability of NIR spectroscopy for the analysis of chemical weapons has been demonstrated for several blister and nerve agents using a portable NIR spectrometer (pNIRS). Spectral data obtained with the pNIRS device were confirmed using a high-resolution benchtop NIR instrument. In addition, density functional theory (DFT) calculations were applied to assess the obtained NIR spectra.

**Figure 1:**
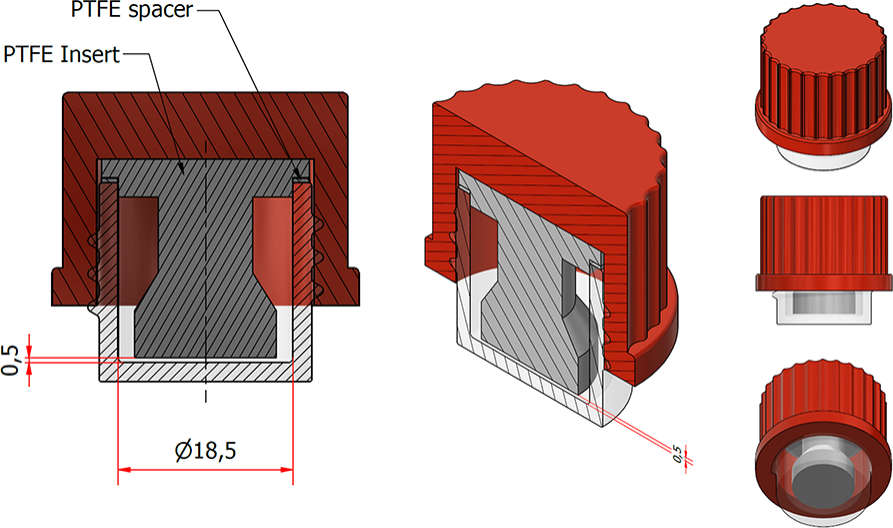
Design of the NIR liquid cell, consisting of a 3D-printed glass base, a PTFE insert, PTFE spacer, and poly butylene terephthalate (PBT) with glass fiber cap to seal the cell. The spacer results in a void of 0.5 mm at the bottom of the cell, allowing the formation of a layer of liquid. Analysis can be done with amounts as little as 50 μL, but typically 100 μL is added for the formation of a consistent liquid layer and reliable analysis.

## Materials and Methods

### Design and Production of the Liquid Cell (3D Printing)

3D printing was done on an Asiga Max X 3D printer (Asiga, Sydney, Australia). Printing designs were made with the Autodesk Inventor software. The glass was 3D-printed using Digital Light Processing. Glassomer SL V2 (Glassomer GmbH, Freiburg im Breisgau, Germany) was used as a resin, a monomer containing 40 nm silica-particles. The resin was polymerized under 385 μm UV light. After printing, the nonpolymerized material was removed. To that end, the glass cell was subjected to a debinding process in which the temperature was gradually increased to 600 °C over 30 h, followed by a sintering step under vacuum at 1300 °C. The final result was quartz glass, in the desired 3D shape and with a high light permeability.^26^ For optimal spectroscopic performance, the bottom of the cell was briefly flame polished. Polytetrafluoroethylene (PTFE) material was purchased from Eriks (Alkmaar, The Netherlands). The PTFE insert and spacer were constructed from the purchased PTFE material using a lathe machine tool at the University of Amsterdam workshop. GL32 screw caps (PBT with glass fiber) were purchased from DWK Life Sciences (Wertheim, Germany), and they were used without any additional modification. The cell after the manufacturing process is depicted in Figure 2.

**Figure 2:**
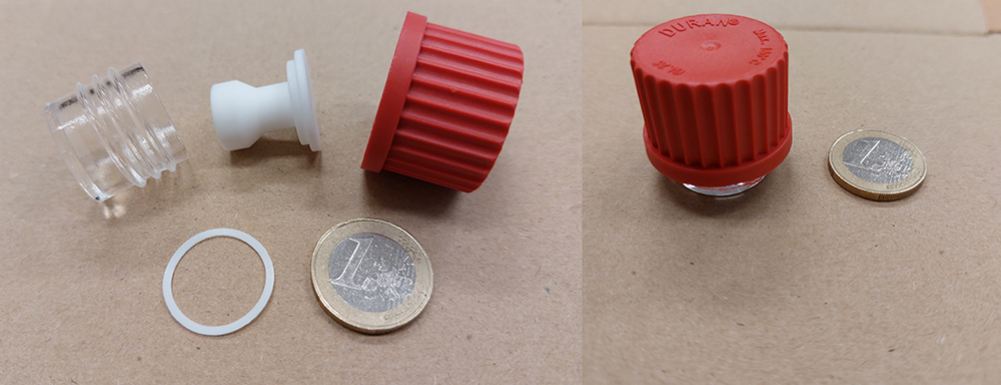
Photos of its individual parts and a fully assembled liquid cell. A euro coin is added as a scale reference. The glass vial, PTFE insert, PTFE spacer, and PBT with glass fiber cap are visible on the left. After liquid is added to the glass container, the insert and spacer are positioned, after which the cap is used to close the cell as depicted on the right.

### Application of the Liquid Cell to CWAs

The nerve agents tabun (GA), sarin (GB), soman (GD), cyclosarin (GF), and VX and the blister agents sulfur mustard (HD), Lewisite 1 (L1), and nitrogen mustard (HN3) are extremely toxic chemicals. Experiments were conducted at the High-Tox facility, which is specially equipped for working with such toxic chemicals. Experimental work was conducted by personnel trained in handling these types of substances. TNO Rijswijk is allowed to handle these chemicals for research purposes. Materials for this study were produced in-house. Chemical identity and purity were confirmed using nuclear magnetic resonance (NMR) and gas chromatography–mass spectrometry (GC-MS) and exceeded 95%. For the sake of public safety, details of the small scale production and testing of CWAs at TNO Rijswijk cannot be shared.

After ensuring that the liquid cells were airtight and yielded high quality NIR spectra using organic solvents, they were applied for CWA identification. For this, the cells were opened, the inserts removed, and 100 μL of the chemical of interest was carefully added. The insert was then slowly placed back in the cell, after which the lid was tightened. This procedure resulted in the formation of a 0.5 mm thick layer of liquid trapped between the glass bottom of the cell and the PTFE insert. As the light reflects back from the insert, this roughly leads to an overall path length of 1 mm, although the light reflection pattern is more complex due to the angle of the three incident NIR light sources from the device. To test for gas leaks, a portable ion mobility spectrometer (LCD 3.3, Smiths Detection) was held at different positions around the cells after tightening the cap.

### Validation Experiments

The 3D glass printing process was expected to result in minimal variation in the produced liquid cells. To test for the effects of cell production variation, a single chemical was repeatedly measured in different cells. The hydrolysis product of sulfur mustard, thiodiglycol (TDG) was selected for this and purchased from Sigma-Aldrich (Zwijndrecht, The Netherlands) with a purity of ≥ 99%. A 100 μL sample of TDG was added to five different cells and analyzed for five repetitions (*n* = 5 × 5), where the cell was physically repositioned on the NIR device between measurements.

The influence of the liquid cell on the stability of the compounds was assessed by storing different CWAs inside liquid cells at room temperature for 41 days. NIR spectra were recorded at days 0, 1, 3, 7, 14, 28, and 41. Five spectra were recorded at each time point for each individual sample where the cell was physically repositioned between measurements to take into account the positional variation in the manual process. For safety, the cells were stored inside a metal container in a fume hood.

### NIR Measurements

NIR measurements were conducted at the High-Tox facility to avoid transportation of the CWAs. NIR measurements were done in transflectance mode, where the NIR light enters the liquid film, is reflected by the PTFE insert, and then migrates once more through the liquid film before being detected in the device. Cells containing a liquid sample were placed on top of the NIR detector so that light exposure occurs from the bottom of the glass cell.

Two different NIR detectors were used: a portable instrument operating in the 1350–2550 nm wavelength range and a high-resolution lab instrument, covering a 350–2500 nm wavelength range. The portable NIR, the Puck (Si-Ware, Cairo, Egypt), equipped with a MEMS sensor, acquired 257 points over this wavelength range with a resolution of 16 nm (FWHM). The ASD LabSpec 4 (Malvern Panalytical, Malvern, UK) was used as a control for the performance of the Puck instrument. This instrument acquired 2151 data points over the wavelength range, with a resolution of 10 nm (FWHM).

Both instruments required a blank or calibration measurement before analysis of the sample to account for the NIR light source variations over the wavelength range. An empty liquid cell was used as a blank for both of the detectors. The portable NIR instrument allowed direct placement of the sample on top of the detector, as visible in Figure S1b of the Supporting Information. The benchtop NIR was operated with the contact probe accessory, on top of which the liquid cell was placed.

### Data Analysis

Data import and processing for the Puck were done in Excel. Data import for the ASD LabSpec 4 data was done in R 4.3.3, using Rstudio 2023.12.1 build 402. The R-Package asdreader was used for this.^32^ Further data processing was done in Excel.

### Computational Methods

Quantum-chemical calculations at the DFT level were performed at the Dutch National supercomputer Snellius. Initial geometries were created from the SMILES code and preoptimized with MMFF94. A conformational search was done with CREST v2.12 using the GFN2-xTB functional.^33^ The resulting conformers were selected up to a relative energy threshold of 40 kJ/mol and then optimized using DFT at the RI-BP86/def2-SVP level as implemented in ORCA 6.0.1 without the use of a solvation model.^34^ For the conformers up to a free energy of 3 kJ/mol, the near IR spectrum was predicted at the same level of theory using the ORCA keyword NearIR, which accounts for anharmonic effects in band intensities (at the xTB level) but not in frequencies. The stick spectrum was convolved with a Gaussian line shape function of 40 cm^–1^ FWHM, after which the spectrum was converted to wavelength (in nm). The wavelength is empirically scaled by dividing by a factor that changes linearly from 0.96 at 1600 nm to 1.0 at 2550 nm to correct for systematic errors in the method. In addition, Gaussian16 was used to calculate anharmonic frequencies and intensities employing GVPT2 at the B3LYP/def2-TZVP level, after geometry optimization (VeryTight) at the same level of theory.^35^ Overtones and combination bands up to three quanta were included using the keyword Spectro = MaxQuanta = 3. The stick spectrum was convoluted with a Gaussian function of 40 cm^–1^ FWHM and is presented without wavelength scaling.

## Results and Discussion

### Design of the Cell

Different designs were initially tested for the liquid cell, and several insert versions were created. An initial straight cylindrical PTFE column design resulted in a smaller available volume in the cell and trapped air pushing out any excess liquid. This led to the current conical design that allows for more liquid to be present inside the cell and creates an additional volume available to the liquid during positioning of the insert.

Different materials were tested to optimize the reflectance inside the liquid cell. For ease of manufacturing, a first design was created by using PTFE tape. This type of tape is often used for airtight sealing when connecting gas tubing. To this end, the surface roughness of this type of tape is increased, which also benefits diffuse reflectance. In addition to PTFE tape, aluminum foil was tested as a reflecting material. Household aluminum foil has two different sides, a rough, matte side and smooth, glossy side, leading to yielding different reflective properties.^36^ Both sides of the aluminum foil showed promising results in terms of reflectance. In initial versions of the cell, aluminum foil and PTFE tape were wrapped around an insert to create the reflecting layer within the cell. Although high quality NIR spectra were obtained, these designs have limitations in terms of robustness and inertness. Aluminum foil forms a layer of aluminum oxide on the outer layer upon contact with oxygen.^37^ This layer can affect the reflective properties observed in the aluminum material. Furthermore, metal oxides among which aluminum oxide, are considered to destructively bind certain CWAs, making them unsuitable for prolonged sample storage.^38,39^ The use of PTFE tape regularly resulted in incomplete sealing and thus liquid loss due to leakage and evaporation, a highly undesirable situation when working with very toxic compounds. Ultimately, it was decided to use the PTFE insert directly as the reflecting surface to ensure robust production and user safety. Although this significantly reduced the overall reflectance, good quality NIR spectra were still obtained for the CWAs.

### Validation Experiment: Reproducibility

The 3D printing of glass proved to be very reproducible, resulting in minimal cell-to-cell variation. Small changes during production can directly affect the spectral data obtained when using the cell. Solid samples often give rise to a variable effective path length depending on the particle properties and arrangement.^40^ Some other effects, such as scattering, can occur in all types of samples.^41^ Liquids are more evenly distributed and more homogeneous than solids, leading to less variation in path length and minimal scattering. After the sample was placed on the NIR detector and the same liquid sample was measured multiple times, almost no variation is observed. Small differences in path length can occur in this instance due to variation in cell production, formation of the liquid layer between the insert and the bottom of the cell, and positioning of the cell on the device. To assess the overall variation and with that the repeatability (on a single NIR device), TDG was measured repeatedly for five different cells. In between the measurements, the cell was repositioned on the detector to include positional variation. Resulting spectra were averaged for each sample cell as depicted in Figure 3.

**Figure 3:**
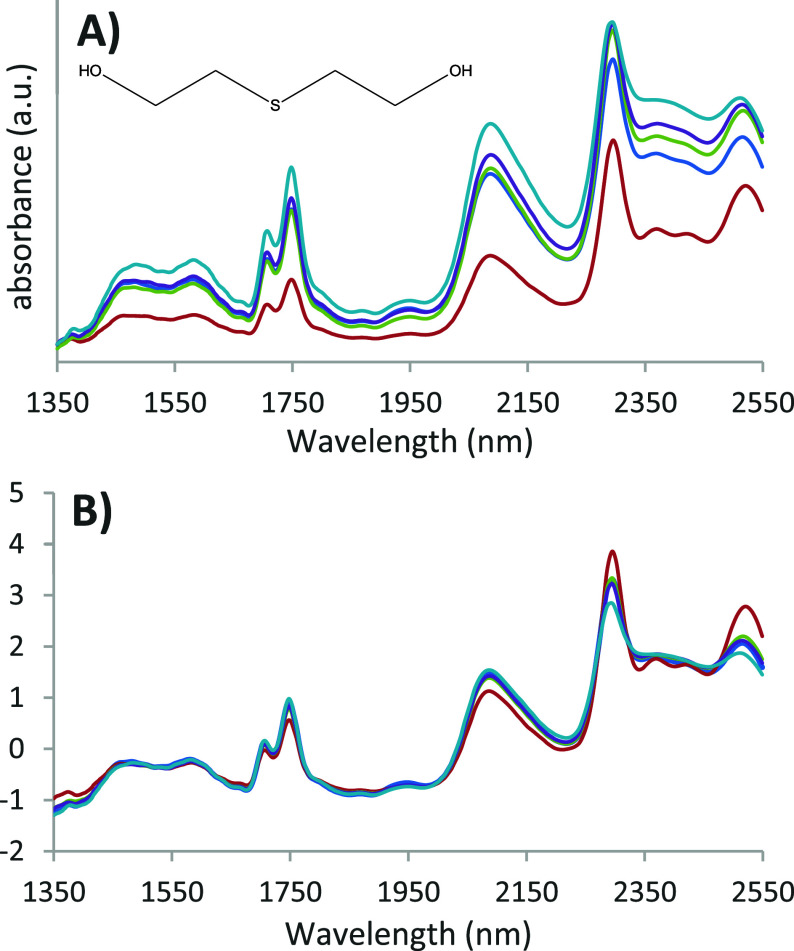
NIR spectra of TDG measured in five different liquid cells. (A) Averaged raw data of five measurements of five different liquid cells. (B) The data after standard normal variate (SNV) preprocessing.

The recorded spectra show little variation in the five measurements from the same cell. A substantial difference is visible in the absolute intensity for the individual cells. Four of the averaged spectra are quite similar in intensity without any preprocessing. The spectrum for one cell (in red) shows a more pronounced deviation in intensity; possibly, this arrangement leads to more efficient reflection of the incident NIR source. After applying a standard normal variate (SNV) preprocessing, a common form of normalization in NIR spectroscopy, cell specific spectral variation becomes minimal. Slight differences can still be seen in the SNV spectra around the 2350–2400 nm wavelength region, with the most pronounced deviation for the cell yielding the spectrum indicated in red. A designated blank cell was used as the calibration cell for all different measurements. No distinct features or wavelength specific signals were observed in the spectra recorded for the empty cells. Therefore, the most obvious explanation is that the observed variability at this wavelength region is caused by a saturation effect, *i.e*., that a too high absorption results in a loss of spectral detail for the other four spectra. For this reason, picking the right layer thickness is important when developing an NIR application. Possibly, an optimal spacer thickness exists for specific compounds, but when dealing with a wide range and/or unknown substances, an intermediate thickness of 0.5 mm as used in this study provides a good compromise between sensitivity and spectral detail. In this respect, developing an insert that provides a higher reflection efficiency could be very beneficial, and further optimization of the insert design is currently ongoing.

The spectra in Figure 3 show that when applying powerful multivariate data classification and identification tools, care has to be taken to prevent false positive and false negative outcomes due to cell-to-cell variations. It is, therefore, recommended to perform quality checks before commissioning newly produced cells. In addition, models should be developed from multicell data sets and after optimal data preprocessing to remove as much cell-to-cell variation as possible. This is especially important given the envisioned single use of cells when sampling dangerous liquids.

### Validation Experiment: Stability

The materials used for the construction of the liquid cells were chosen based on several features, among which chemical inertness was of key importance. As both glass and PTFE are typically inert, it was not expected that any interaction between the chemicals added to the liquid cell and the cell constituents would occur. However, chemical warfare agents are known to be highly reactive and prone to hydrolysis.^42,43^ Therefore, stability measurements were conducted in which the entire range of CWAs were stored in the cells over a prolonged period of time. At specific time intervals, NIR measurements were conducted to assess sample stability using the NIR spectrum. Two examples of the stability experiment are shown in Figure 4 (results of other stability measurements can be found in the Supporting Information, Figures S10–S15).

**Figure 4:**
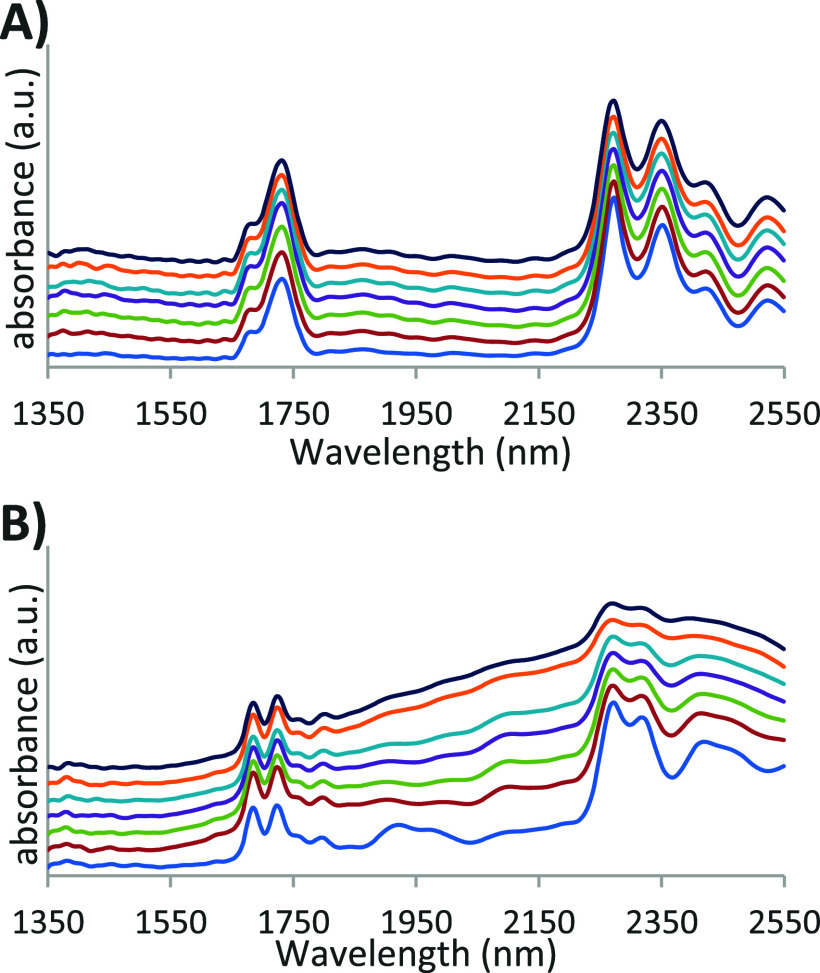
Stability measurements of sulfur mustard (A) and sarin (B). An offset was manually added to the spectra for visualization. In both cases, spectra are shown in chronological order using a spectral offset, starting from bottom to top: 0, 1, 3, 7, 14, 28, and 41 days.

Sarin is known for its limited stability and rapid degradation once released in the environment,^44^ while sulfur mustard is known to be quite stable for a CWA. This was also confirmed in the current experiment. Figure 4A demonstrates the spectral integrity of sulfur mustard over the entire storage period. But while the two sarin features around 1700 nm remain persistent, the spectral details in the 2200–2500 nm range slowly fade over time. Sarin is known to hydrolyze into isopropyl methylphosphonic acid (IMPA), which has a broad NIR signal with less distinct features (see Figure S9 of the Supporting Information for a spectrum of the reference standard). The spectrum of sarin slowly transforms into that of IMPA, indicative of the presence of both the agent and its hydrolysis product until after 41 days, only IMPA remains. Similar behavior was observed for soman, as shown in Figure S15 of the Supporting Information. All measurements between the start and end of the time series show a mixed spectrum that is composed of the reference spectra of pure sarin and IMPA. This indicates that it might be feasible to measure and identify mixtures of two or more compounds. This has already been proven using the Puck on solid samples and is currently investigated by our team for CWA liquids.^14^ For partially hydrolyzed samples, it would be possible to detect the original CWA and its hydrolysis product by using, for instance, a partial least squares (PLS) model, which would even allow an estimation of the degree of hydrolysis on the basis of the relative contribution to the overall NIR spectrum. The excellent spectral stability of sulfur mustard demonstrates that the liquid cell does not lead to specific compound loss or degradation. This indicates that samples can be analyzed for a prolonged period of time after sampling. It is expected that this period can be further extended for some compounds when samples are stored in a fridge or freezer.

During the experiments, an IMS was used to check for any chemicals leaking from the cells. The cells containing the CWAs were kept in a fume hood, and the IMS was held at different locations surrounding the cell shortly after adding the liquids. No leaks were observed at this stage. When the liquid cells were stored inside a closed metal container during the stability experiments, IMS measurements were repeated inside the metal container. While measuring in this closed environment, small amounts of sarin could be observed using IMS. None of the other chemicals could be detected this way. This means that even though the containers are found to be tight for liquids, small amounts of gas might still escape in the case of highly volatile compounds. While in circumstances where samples are taken in the field, personnel is likely in protective clothing and more dangerous vapors are around, potential leakage should be taken into account when dealing with chemicals of such high toxicity. As an additional security precaution, cells should therefore be placed in a separate, larger container and should be stored under adequate ventilation, e.g., in a fume hood. Furthermore, migration of air, moisture, and other contaminants into the cell might affect the stability of the sample as this could catalyze the degradation and hydrolysis of certain agents.^45^

Even though several of the tested chemicals are quite volatile, evaporation was minimal and no visible changes were observed in the amount of liquid present in the cells. Enough volume was still present after 41 days to successfully measure the samples.

### Identification of CWAs and Comparison to a Benchtop NIR Instrument

The Puck is a portable NIR device with a lower spectral resolution compared to that of a high-end benchtop instrument. As no spectra of CWAs have previously been published in the open literature, no comparison can be made for the obtained spectra to assess the correctness of the field data. A high-resolution instrument, ASD LabSpec 4, was used to measure the same sample set. Spectra of both the Powder Puck and the ASD LabSpec 4 for sarin, VX, sulfur mustard, and nitrogen mustard are shown in Figure 5. On the basis of the excellent spectral agreement, it can be concluded that the rapid, on-site use of the Puck will yield accurate NIR transflectance spectra of CWA liquids in the wavelength range covered.

**Figure 5:**
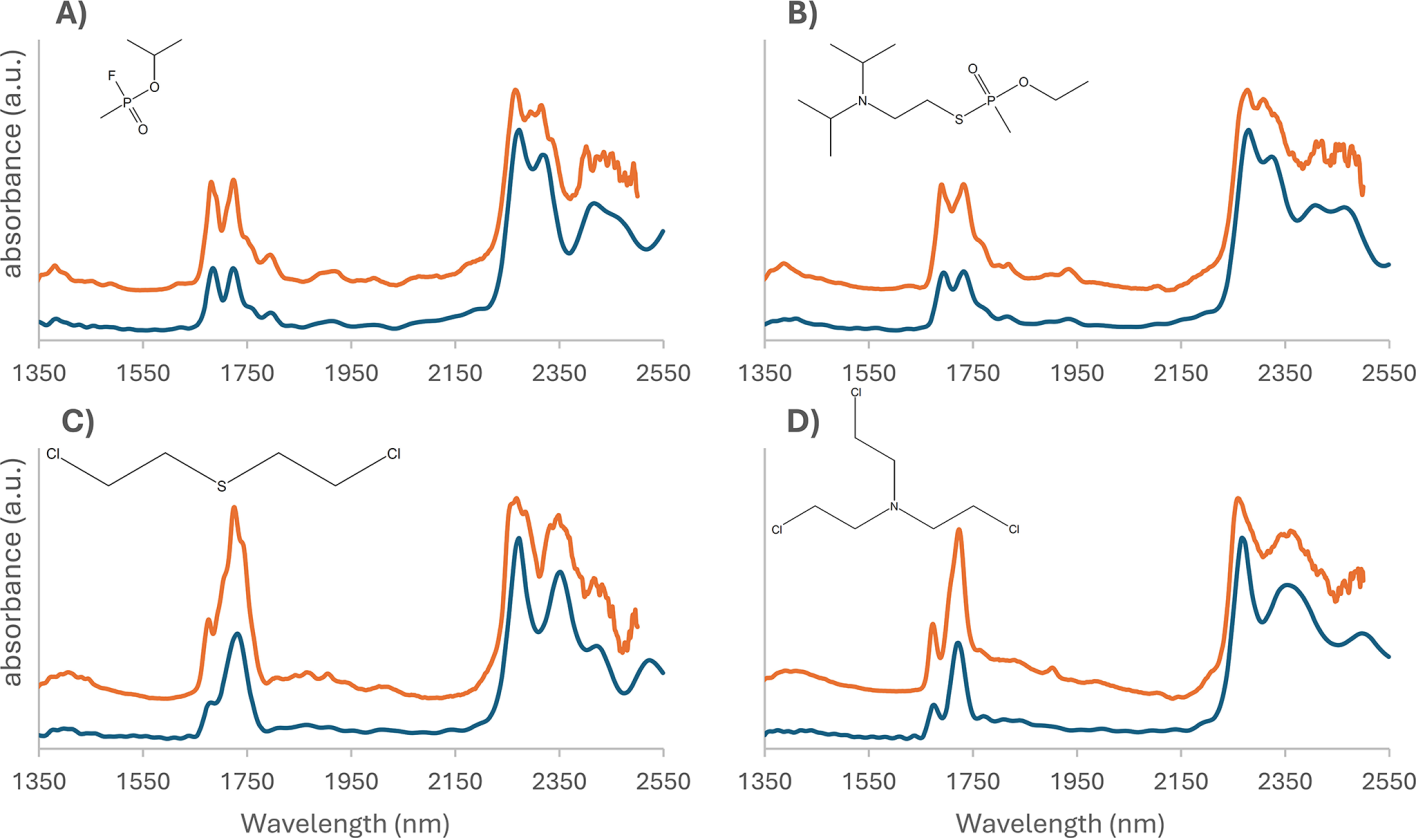
NIR spectra of (A) sarin, (B) VX, (C) sulfur mustard, and (D) nitrogen mustard (HN3) using two different NIR instruments. The spectra in blue were recorded with the Puck, and the spectra in orange were recorded with the ASD LabSpec 4. The offset was manually created for visualization purposes.

However, the higher resolution of the ASD instrument leads to more distinct minor features in the NIR spectrum that are not visible when using the Puck device. Overall, the pattern of the spectra and the wavelength position of the features are the same. The ASD instrument covers a more extended spectral range, starting at 350 nm in the UV/Vis domain and ending at 2500 nm. The full ASD spectra for sarin and sulfur mustard are displayed in Figures S6 and S7, respectively, of the Supporting Information. In line with results recently reported for drugs of abuse,^15^ these figures illustrate that most compound-specific NIR spectral detail can be found in the wavelength range covered by the Puck device,

Sarin and VX are both organophosphorus nerve agents. While there is structural similarity, they are from a different series of nerve agents. Sarin belongs to the so-called G-Series, while VX belongs to the V-Series.^43^ Sulfur mustard and nitrogen mustard are both classified as blister agents, and these compounds share chemical features such as the presence of chlorine. In contrast to sulfur mustard, nitrogen mustard contains an ethylamine group.^46^

NIR spectra consist of different regions of overtones with single functional group signals repeatedly returning in the wavelength region below 2100 nm. The higher wavelength region contains signals from combination band vibrations.^47^ While it is difficult to directly interpret NIR spectra, visual comparison shows clear differences in the spectra of these chemical agents in both the overtone regions and the combination band regions. This results in characteristic spectra for all CWAs that can be used to detect and identify threat agents in the field using a portable NIR device and a suitable reference library. Nerve agents within the same series are structurally more closely related, making the distinguishing between them more difficult. However, clear differences are visible in the measured spectra of the four nerve agents of the G-Series (GA, GB, GD, and GF). Full spectra of GA, GD, and GF are depicted in Figure S8 of the Supporting Information. As expected, there is a larger visual difference in the spectra of the blister agents and the nerve agents than between compounds of the same class. Consequently, the spectra can possibly also be used to appoint a CWA to a certain class. This could be useful when encountering a novel compound for the first time and in the absence of a library reference spectrum.

### DFT Calculation of CWA NIR Spectra

DFT calculations were undertaken to theoretically predict the NIR spectra of the different CWAs. Figure 6 displays, as an example, the experimentally measured spectrum of sarin and its predicted spectrum. The main features in the experimental NIR spectrum around 1700 nm and at wavelengths exceeding 2200 nm are well reproduced by the calculations. The 1700 nm absorptions relate to C–H stretch overtones and features at longer wavelength originate from combinations of C–H stretching and bending vibrations. Organophosphates with a free OH group show a broad baseline absorption that increases with wavelength. This broad band is well-known for intermolecular O–H hydrogen-bonding interactions, spreading out to 2800 nm for phosphinic acids.^48,49^ While phosphonic acids have not been as extensively studied for DFT calculations of NIR spectra, they are structurally similar to phosphinic acids and capable of forming similar intramolecular O–H interactions.^50^ The dynamic behavior of such hydrogen-bonded moieties is not accounted for by the static (anharmonic) DFT calculations, so that the measured spectrum cannot be reproduced.

**Figure 6:**
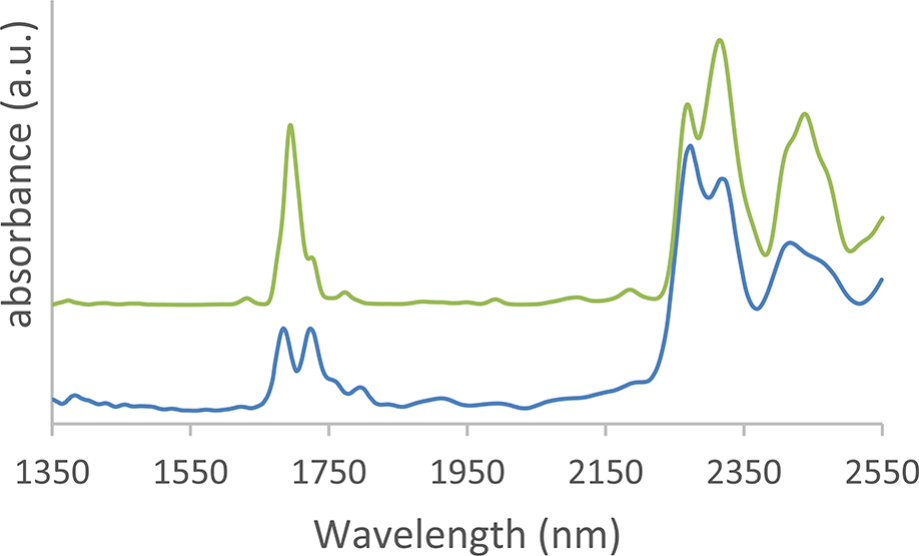
Comparison of DFT calculated (green) and experimental NIR spectra (blue) of sarin. The experimental spectrum is measured with the Puck. The calculated anharmonic spectrum was obtained using the GVPT2 method at the B3LYP/def2-TZVP level. The vertical offset was manually added for visualization.

The ORCA NearIR calculations can predict the main peaks in the spectra and their relative intensities. The GVPT2 calculations compute force constants to fourth order, allowing one to also predict 3-quantum combinations, which explain minor features, for instance, seen at wavelengths shorter than 1600 nm and between 1800 and 2200 nm. Furthermore, the relative intensities are better predicted by a higher level of theory.

## Conclusions

The proposed 3D-printed glass cell design for the NIR characterization of liquids allows for rapid and on-site analysis of highly dangerous CWAs. The design is safe to handle and results in information-rich, molecule-specific spectra that can be used to identify threat agents. The 3D printing of glass results in minimal variation in the product specifications. The production of a cell costs less than 20 euros in material and is considered low enough for the cells to be applied as a single use sample container, as this further ensures operator safety. In addition, the use of the cell circumvents detector contamination by containing the liquid within the cell, avoiding time consuming and costly decontamination procedures. Validation experiments show that data can be recorded in a reproducible manner by using different cells. This enables compound identification on the basis of reference libraries and the use of multivariate data analysis methods to predict the compound class and unravel mixture compositions. The stability of several CWAs in the liquid cell was investigated and showed no enhanced degradation of the agents in the cell environment. The cell has the potential to be used as an on-site sampling device, which after transfer of the liquid to the cell allows for safe handling, analysis, and transport back to an expert laboratory for confirmatory analysis. Before use in practice, variable environmental conditions, such as temperatures and humidity, should be further tested to ensure sample integrity under different operational circumstances. In addition, it should be noted that, while not included in the current work, some CWAs like Novichok analogues might interfere with glassware, which may not affect stability directly but may complicate subsequent analysis using other techniques. The application of the liquid cell is expected to be suitable for many other fields dealing with liquid sample matrices.

Spectra of several CWAs were recorded using both a portable and benchtop laboratory NIR device. Recorded spectra proved to be very similar, although the benchtop instrument shows more spectral detail due to its higher resolution. The designed cell for NIR liquid analysis can be used on different instruments, broadening its application scope. The recorded spectra of the CWAs displayed significant differences in various regions of the NIR spectrum, allowing for the confident detection and identification of CWAs. The portability of the NIR devices enables rapid on-site analysis in different scenarios. However, intact, relatively pure CWAs are not frequently encountered, and currently, our research efforts focus on the identification of threat agents in more realistic but also more complex sample matrices. To this end, complex mixture compositions and lower amounts of chemicals in various soil types are currently being investigated. Before broader application is feasible, the sensitivity and selectivity of the technique under different scenarios should be thoroughly established under changing operational conditions. Additionally, identification models should be carefully constructed, taking into account possible mixtures and interferents. While CWA analysis using on-site NIR spectroscopy cannot be broadly applied as of yet, the current methodology could already be useful in situations where intact liquids are encountered. This study provides the tools and lays the foundation to develop safe, rapid, and robust NIR-based CWA analyses in more realistic scenarios.

DFT calculations were performed on several CWAs and related compounds to add confidence in the obtained spectra using NIR. The calculation of NIR spectra is found to be quite complex; the peak position can be relatively accurately predicted, but peak intensity is computationally intensive and gives rise to differences with experimentally observed spectra. Nonetheless, a satisfactory level of agreement was observed. If the DFT prediction of NIR spectra becomes more accurate and more easily accessible, this could become a promising tool in the structure elucidation of unknowns on the basis of a measured NIR spectrum.

## Supplementary Material



## Data Availability

The .ipt file for printing of the liquid cell is available at https://doi.org/10.5281/zenodo.17091829.

## Notes

Additional images of the liquid cell, images of the manufacturing process of the liquid cells, and full range and additional spectra of the different CWAs and IMPA. Spectra of the stability study (PDF)
