# Supplementary material for: Rapid On-Site NIR Spectroscopic Characterization of CWA Liquids Using a Novel 3D-Printed Glass Cell
Source: Anal Chem. 2025 Sep 16;97(38):20973–81. doi: 10.1021/acs.analchem.5c03719 (PMC12489889; doi:10.1021/acs.analchem.5c03719)
Supplement: Supplementary file 1 [file ac5c03719_si_001.pdf]

Supporting Information

Rapid On-Site NIR Spectroscopic Characterization of CWA Liquids Using a Novel 3D-Printed Glass Cell

Jelle C. de Koning[a,b]*, Marcel J. van der Schans[a], Lai Fun Chau[a], Tom Venema[a], Gert IJ. Salentijn[d,e], Saer Samanipour[b], Gertjan Bon[f], Henk-Jan Ramaker[g], Teun van Wieringen[f], Jos Oomens[f], Arian C. van Asten[b,c]

[a] TNO Defence Safety and Security, Dep. CBRN Protection, Lange Kleiweg 137, 2288GJ Rijswijk, The Netherlands
[b] van 't Hoff Institute for Molecular Sciences, Faculty of Science, University of Amsterdam, P.O. Box 94157, 1090 GD Amsterdam, The Netherlands
[c] CLHC, Netherlands Center for Forensic Science and Medicine, University of Amsterdam, P.O. Box 94157, 1090 GD Amsterdam, The Netherlands
[d] Laboratory of Organic Chemistry, Wageningen University & Research, Stippeneng 4, 6708 WE Wageningen
[e] Wageningen Food Safety Research, P.O. Box 230, 6700 AE Wageningen, The Netherlands
[f] Technology Centre, Faculty of Science, , University of Amsterdam, P.O. Box 94157, 1090GD Amsterdam, The Netherlands
[g] TIPb, Wilhelminaplein 30, 1062 KR Amsterdam, the Netherlands
[h] FELIX laboratory, Institute for Molecules and Materials, Radboud University, Toernooiveld 7, 6525 ED Nijmegen, The Netherlands
* Corresponding author

Table of contents

Page S2: Figure S1a - Design of the NIR Liquid Cell with measurements of the cell, insert and spacer

Figure S1b – Photo and schematic overview of position of the liquid cell on the Puck

Page S3 Figure S2 – Liquids cells during the printing process

Figure S3 - 3D Printed liquid cells after the printing process, cells have an orange color and are slightly larger than the final product

Page S4 Figure S4 - 3D Printed liquid cells after the printing process, during the debinding process. Color changes from orange to white

Figure S5 - Final 3D printed product, a quartz glass liquid cell

Page S5 Figure S6: Full spectrum of Sarin recorded using both NIR instruments

Figure S7: Full spectrum of Sulfur mustard recorded using both NIR instruments

Page S6 Figure S8: Spectra of lewisite, tabun, cyclosarin and soman recorded using both the Puck and the ASD LabSpec 4 instrument

Figure S9: Spectrum of sarin hydrolysis product IMPA

Page S7 Figure S10: Spectra of stability study of Lewisite

Figure S11: Spectra of stability study of Nitrogen Mustard

Figure S12: Spectra of stability study of Tabun

Page S8 Figure S13: Spectra of stability study of Cyclosarin

Figure S14: Spectra of stability study of VX

Figure S15: Spectra of stability study of Soman

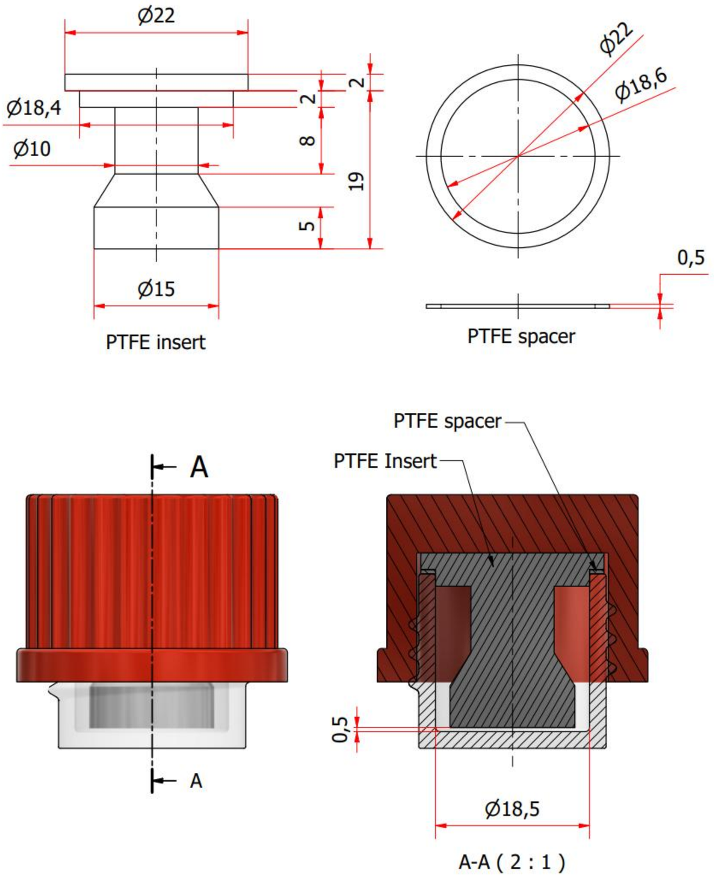

Figure S1a: Design of the NIR Liquid Cell with measurements of the cell, insert and spacer. Both the Insert and spacer are manufactured from polytetrafluoroethylene (PTFE) material. The spacer thickness is determining the size of the void between the PTFE insert and the glass bottom of the liquid cell. By adding a different spacer thickness, the layer thickness for the analysis can be in- or decreased. The effective pathlength roughly equals twice the spacer thickness.

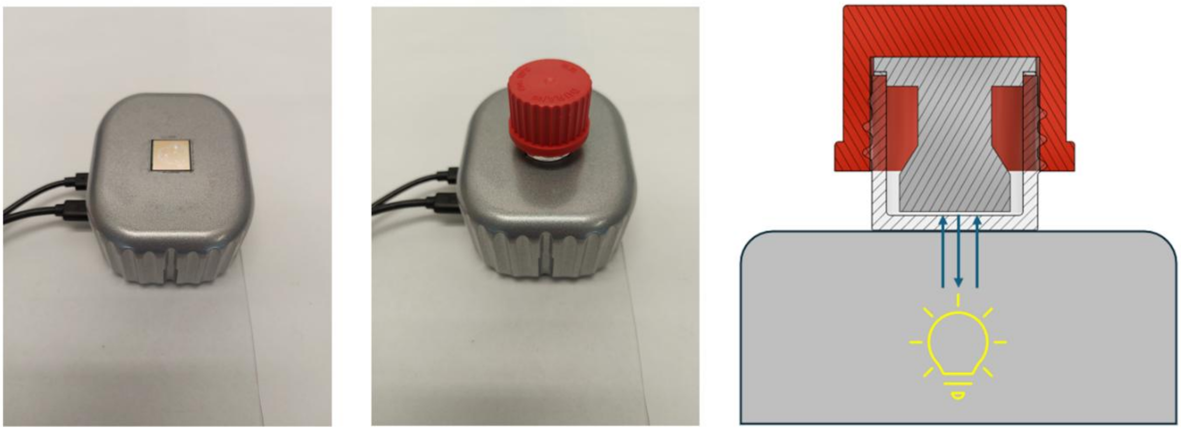

Figure S1b: Photo of the Puck, photo of the liquid cell on the Puck and schematic overview of the liquid cell on the Puck, indicating the position of the light source and the path of the light.

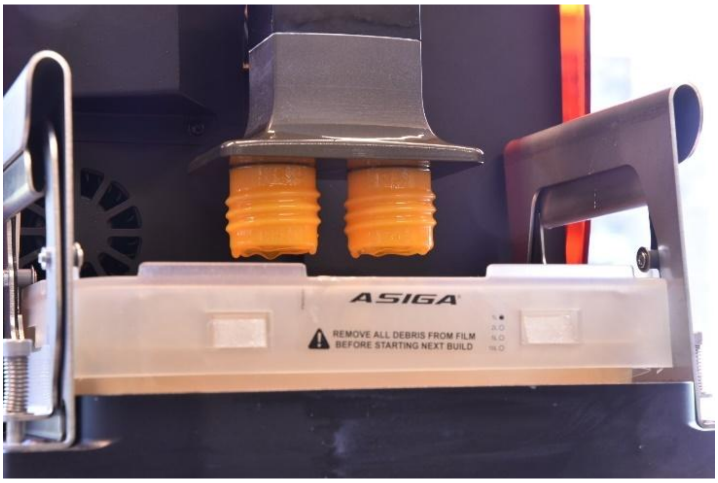

Figure S2: Two of the liquid cells on the 3D printer. Cells are printed using digital light processing where the resin is polymerized under 385 µm UV light. After debinding and sinthering the color is gone and transparent quartz glass is formed, suitable for use in spectroscopy.

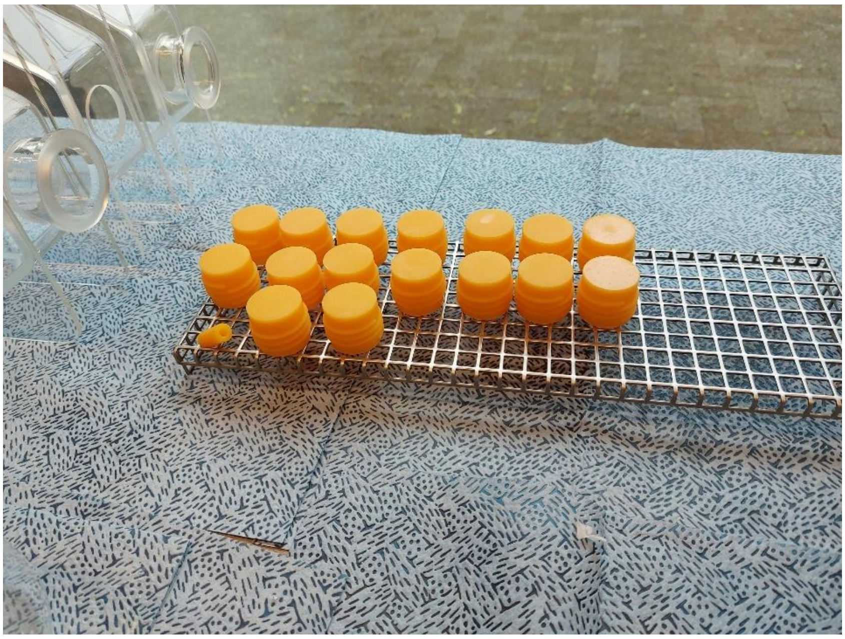

Figure S3: 3D Printed liquid cells after the printing process and removal of excess material. The cells have an orange color originating from the polymer matrix and are slightly larger than the final product.

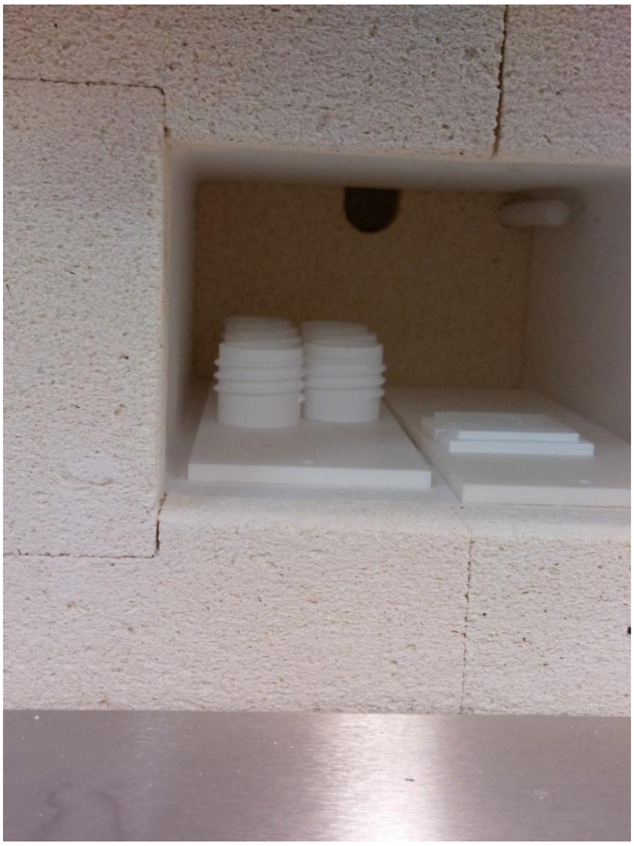

Figure S4: 3D Printed liquid cells after the debinding process where the polymer is thermally removed and the color changes from orange to white.

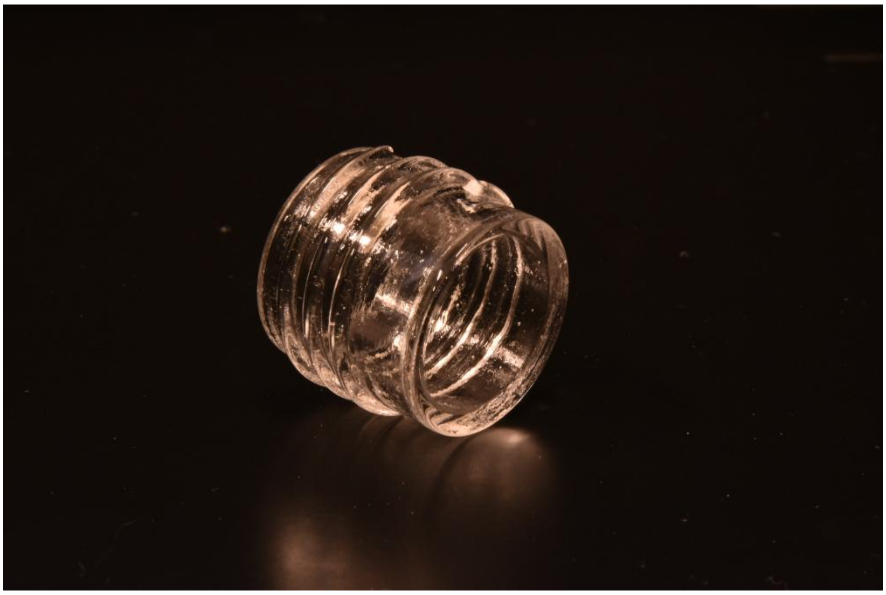

Figure S5: Final 3D printed product after high temperature treatment to form a quartz glass liquid cell. The bottom of the cell is briefly flame-polished.

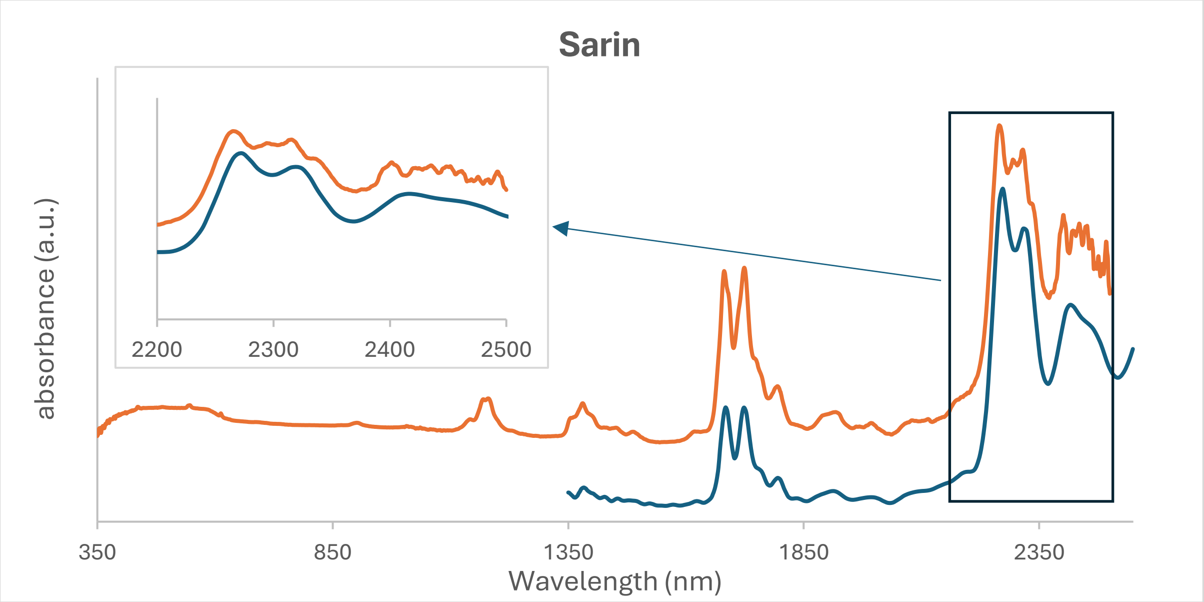

Figure S6: Full spectrum of Sarin recorded using both the Puck and the ASD LabSpec 4 instrument. The blue trace was recorded using the Puck. The orange trace was recorded using the ASD instrument. Most information is captured in the region between 1550-2500 nm of the spectrum. Due to the higher resolution more spectral details are captured with the ASD LabSpec 4. The insert visualizes the difference between both detectors in the 2200-2500 nm area of the spectrum.

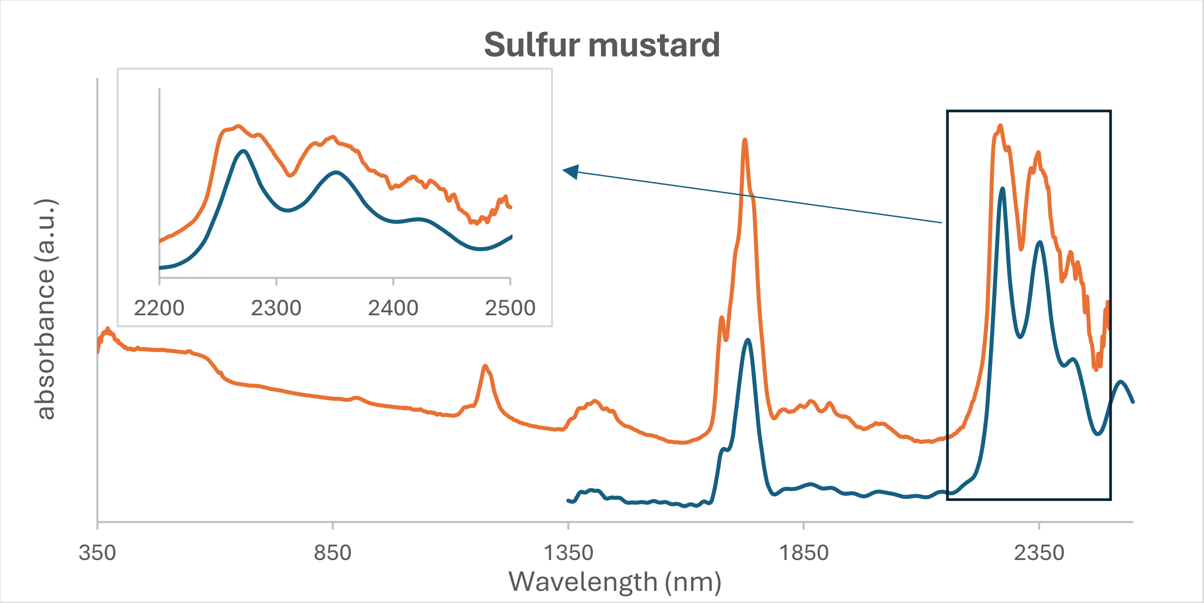

Figure S7: Full spectrum of Sulfur mustard recorded using both the Puck and the ASD LabSpec 4 instrument. The blue spectrum was recorded using the Puck. The orange spectrum was recorded using the ASD instrument. Most information is captured in the region between 1550-2500 nm of the spectrum. Due to the higher resolution more spectral details are captured with the ASD LabSpec 4. The insert visualizes the difference between both detectors in the 2200-2500 nm area of the spectrum.

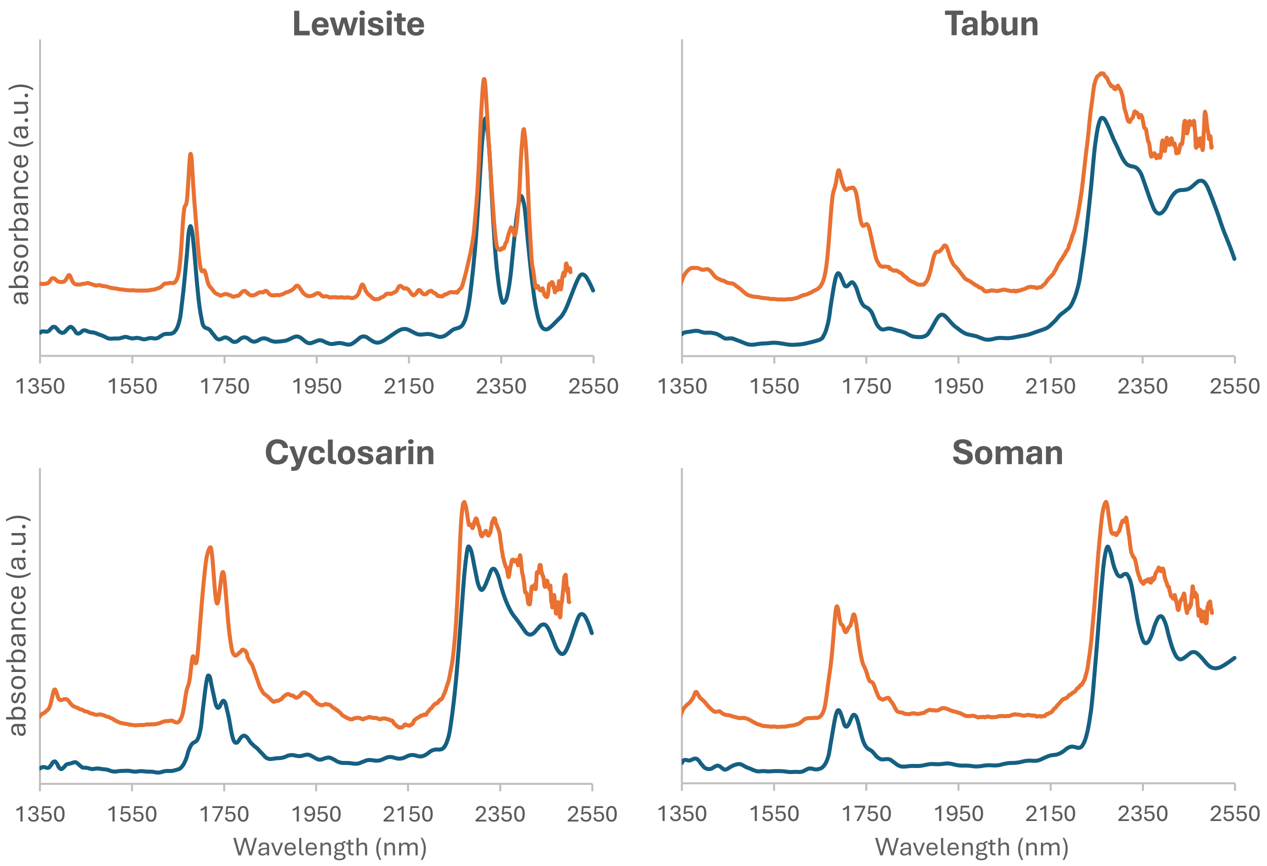

Figure S8: Spectrum of lewisite, tabun, cyclosarin, and soman recorded using both the Puck and the ASD LabSpec 4 instrument. The blue trace was recorded using the Puck. The orange trace was recorded using the ASD instrument.

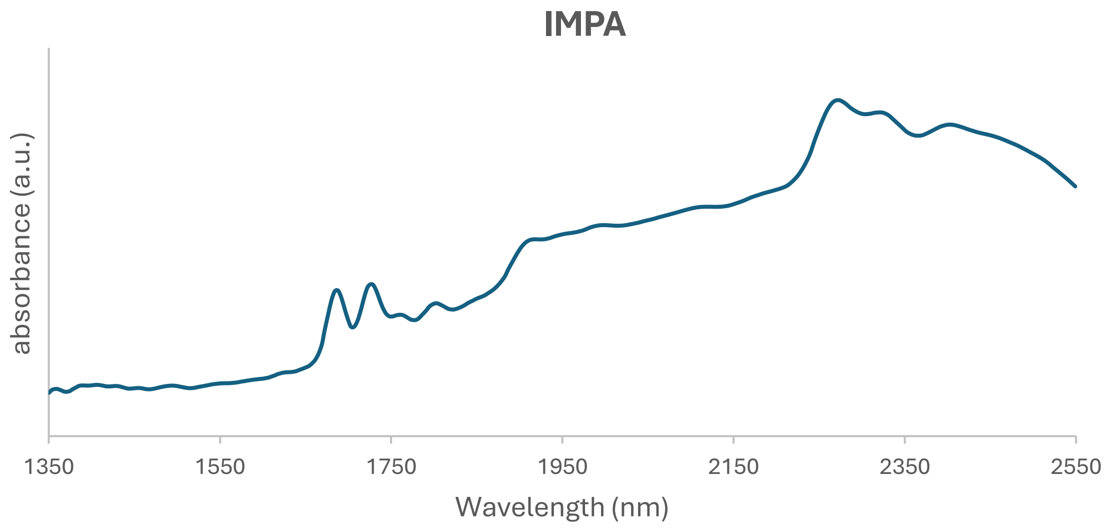

Figure S9: NIR spectrum of sarin hydrolysis product IMPA recorded using the Puck. The broader and less distinct features are caused by the OH group in the molecule.

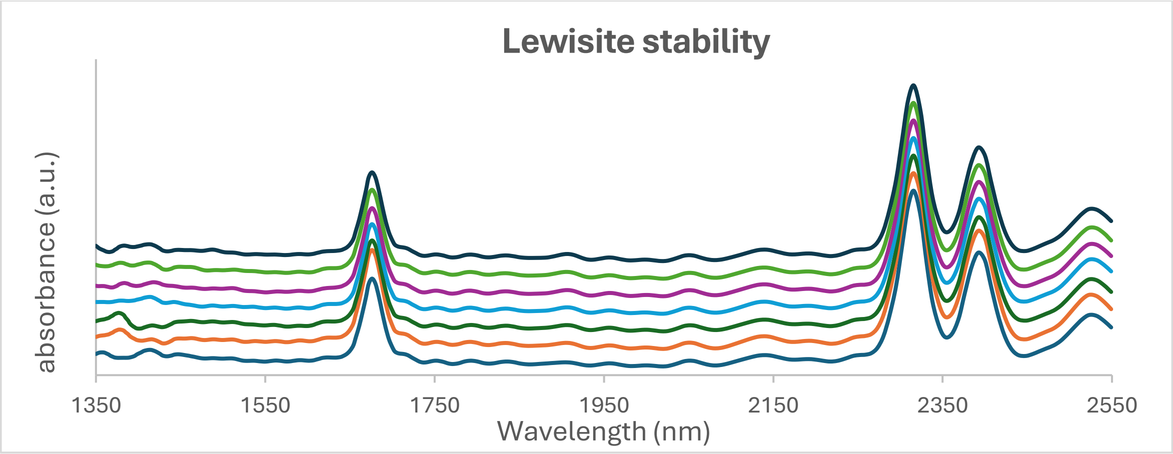

Figure S10: Stability measurements of lewisite. The off-set was manually added for visualization. Spectra are depicted in a chronological order from bottom to top: 0, 1, 3, 7, 14, 28 and 41 days.

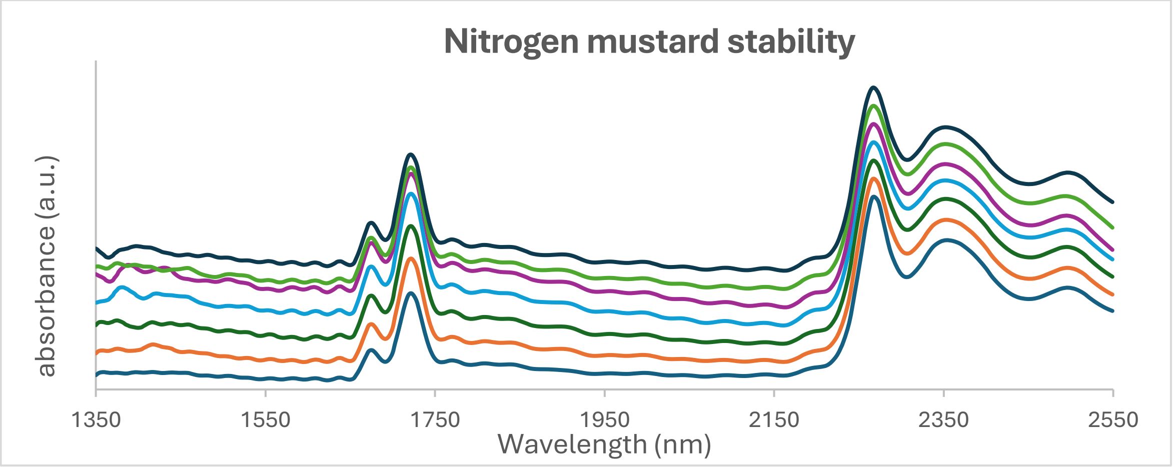

Figure S11: Stability measurements of nitrogen mustard. The off-set was manually added for visualization. Spectra are depicted in a chronological order from bottom to top: 0, 1, 3, 7, 14, 28 and 41 days.

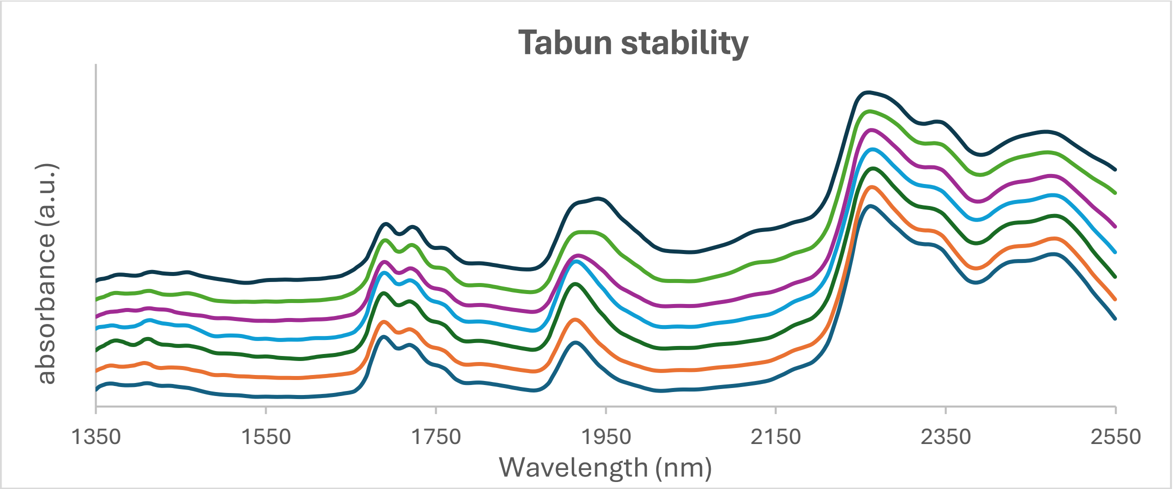

Figure S12: Stability measurements of tabun. The off-set was manually added for visualization. Spectra are depicted in a chronological order from bottom to top: 0, 1, 3, 7, 14, 28 and 41 days.

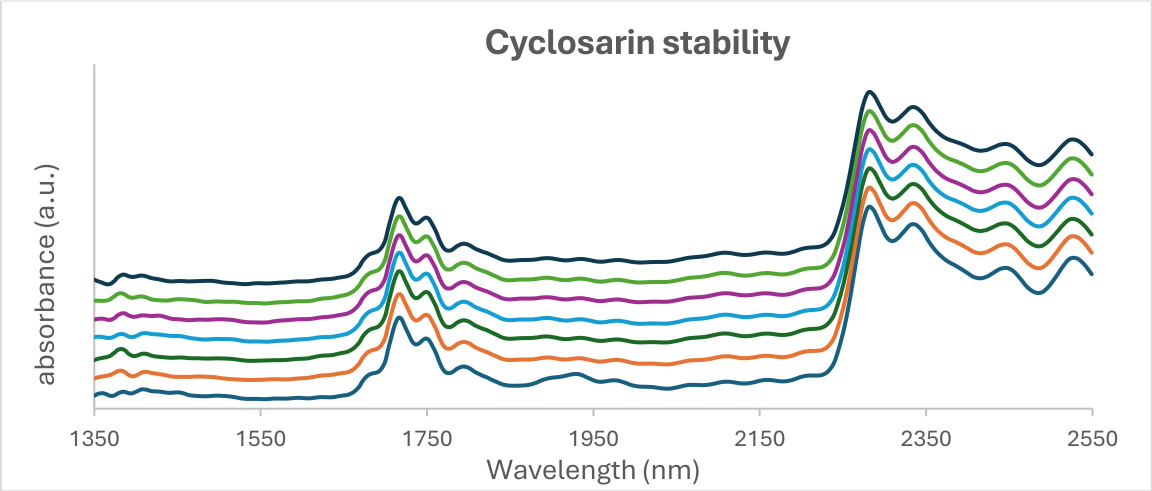

Figure S13: Stability measurements of cyclosarin. The off-set was manually added for visualization. Spectra are depicted in a chronological order from bottom to top: 0, 1, 3, 7, 14, 28 and 41 days.

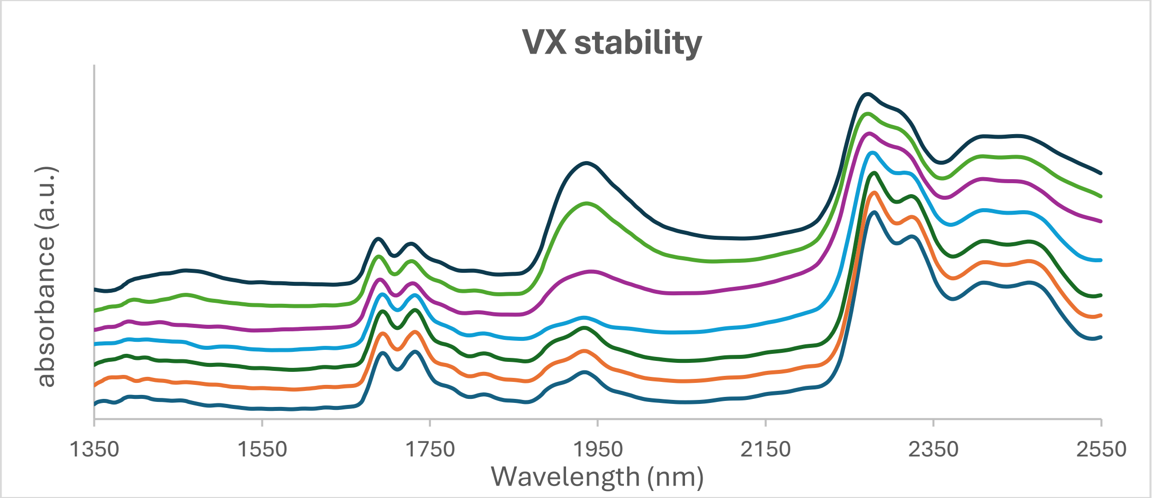

Figure S14: Stability measurements of VX. The off-set was manually added for visualization. Spectra are depicted in a chronological order from bottom to top: 0, 1, 3, 7, 14, 28 and 41 days.

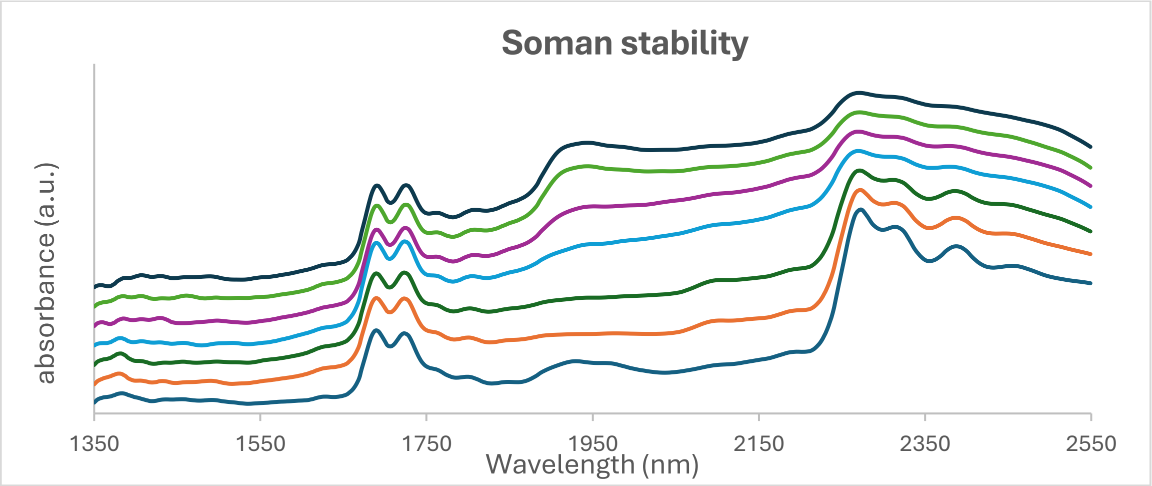

Figure S15: Stability measurements of soman. The off-set was manually added for visualization. Spectra are depicted in a chronological order from bottom to top: 0, 1, 3, 7, 14, 28 and 41 days.
